# Scoliosis in Goldenhar syndrome with curve reversal during brace treatment: a case report

**DOI:** 10.1186/s12891-020-03719-y

**Published:** 2020-10-16

**Authors:** Masashi Uehara, Shugo Kuraishi, Shota Ikegami, Hiroki Oba, Takashi Takizawa, Ryo Munakata, Terue Hatakenaka, Tetsuhiko Mimura, Jun Takahashi

**Affiliations:** Department of Orthopaedic Surgery, Shinshu University School of Medicine, 3-1-1 Asahi, Matsumoto, Nagano, 390-8621 Japan

**Keywords:** Scoliosis, Goldenhar syndrome, Brace, Radiological findings, Overcorrection, Curve reversal

## Abstract

**Background:**

Goldenhar syndrome sometimes displays progressive scoliosis and other spinal deformities that require treatment. However, few reports exist on scoliosis correction in Goldenhar syndrome. We described the rare radiological outcomes of a patient with Goldenhar syndrome who received brace treatment for scoliosis.

**Case presentation:**

A 4-year-old boy was diagnosed as having Goldenhar syndrome and referred to our hospital for scoliosis treatment. The deformity deteriorated gradually, and left convex scoliotic angle was 26 degrees (T3-L2) at 11 years of age. Unexpectedly during treatment with an orthopedic brace, the curve had reversed to 21 degrees (T5-L2) at 7 months of therapy. After another adjustment of the brace, his right convex scoliotic angle improved to 13 degrees (T4-L2) at 15 months of treatment.

**Conclusions:**

Curve reversal may occur during brace treatment for scoliosis in Goldenhar syndrome. Clinicians may opt to periodically check curve correction despite the risk of increased radiation exposure.

## Background

Goldenhar syndrome is a hereditary disease first reported in 1952 as a disorder with facio-auriculo-vertebral dysplasia [1]. The severity of dysplasia varies by case [2, 3]. The frequency of Goldenhar syndrome is estimated at 1 in 3500 to 5600 live births [4, 5]. The male: female ratio of patients is approximately 3:2 [4, 5]. Scoliosis and other spinal deformities have been associated with this syndrome and can sometimes be progressive and require treatment [6]. Vertebral abnormalities and congenital spinal deformities are also common in Goldenhar syndrome [7–9].

To date, there are a few reports on the treatment of scoliosis in Goldenhar syndrome. We herein describe the rare radiological outcomes of an afflicted patient who received a brace for scoliosis correction.

## Case presentation

This study was approved by the institutional ethical review board of Shinshu University School of Medicine (No. 4847) prior to its start and was conducted in accordance with the ethical standards set forth in the 2013 Declaration of Helsinki for research involving human subjects. Written informed consent was obtained from the patient’s parent for publication of this Case report and any accompanying images.

**Fig. 1:**
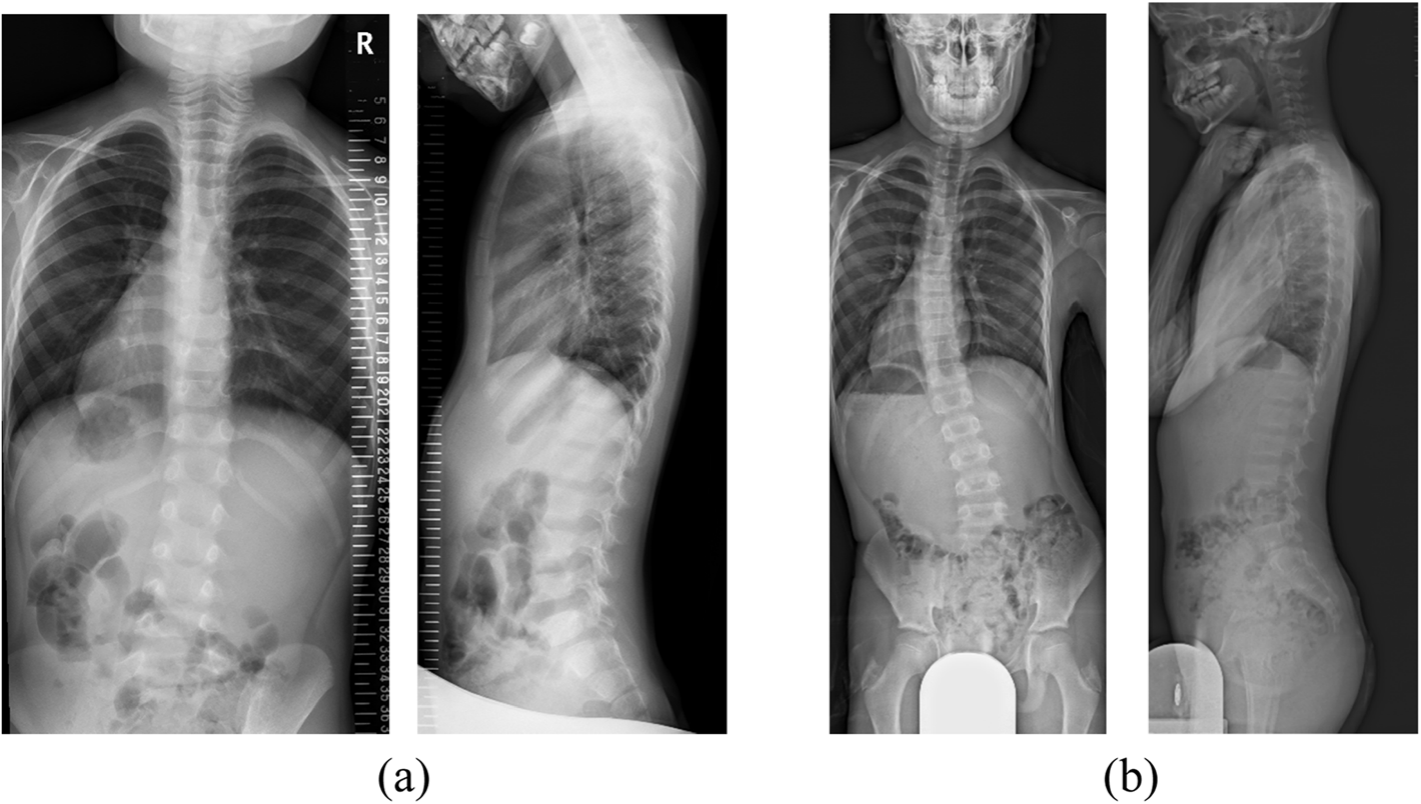
Case: a 4-year-old boy. **a** Scoliotic angle was 5 degrees (T10-L3). **b** Scoliosis deteriorated gradually, and scoliotic angle had increased to 26 degrees (T3-L2) at 11 years of age

**Fig. 2:**
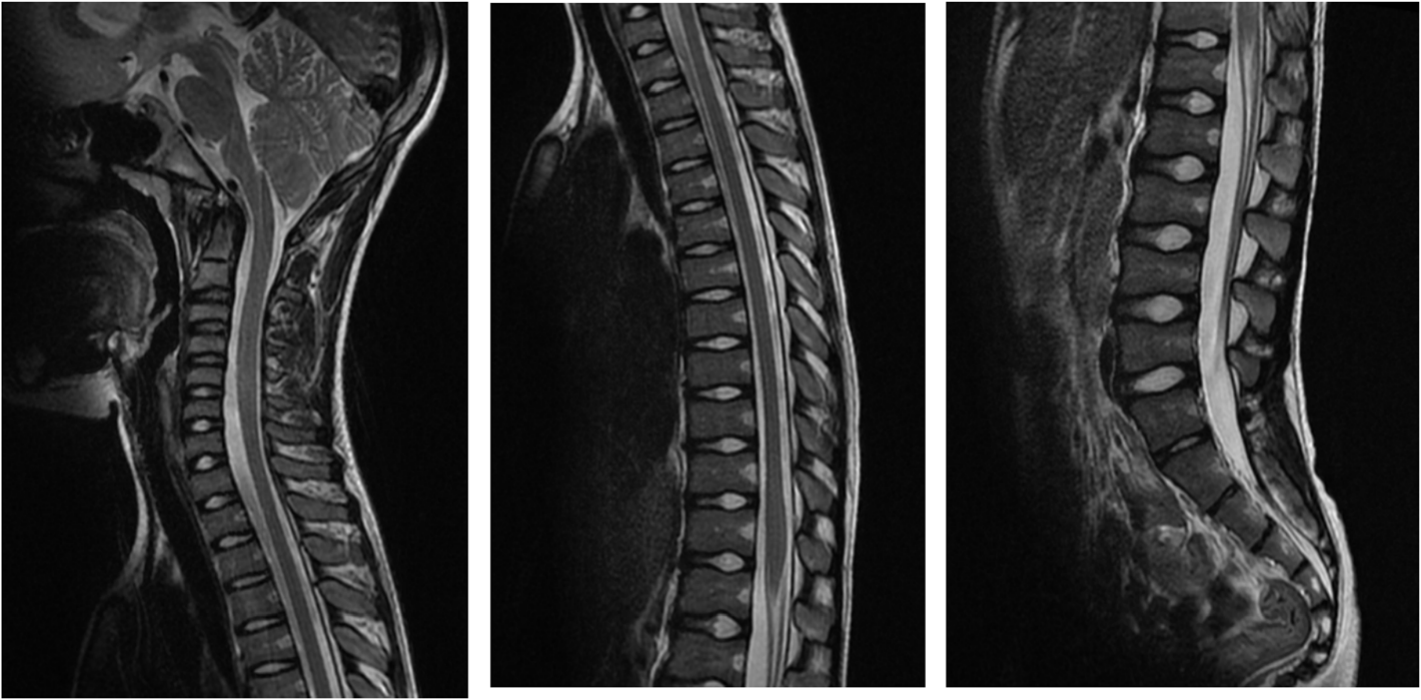
Whole-spine MRI. Whole-spine MRI revealed no abnormalities, such as Chiari malformations, syringomyelia, spinal cord cavities, spinal cord tumors, dural ectasia, and low-level conus medullaris

**Fig. 3:**
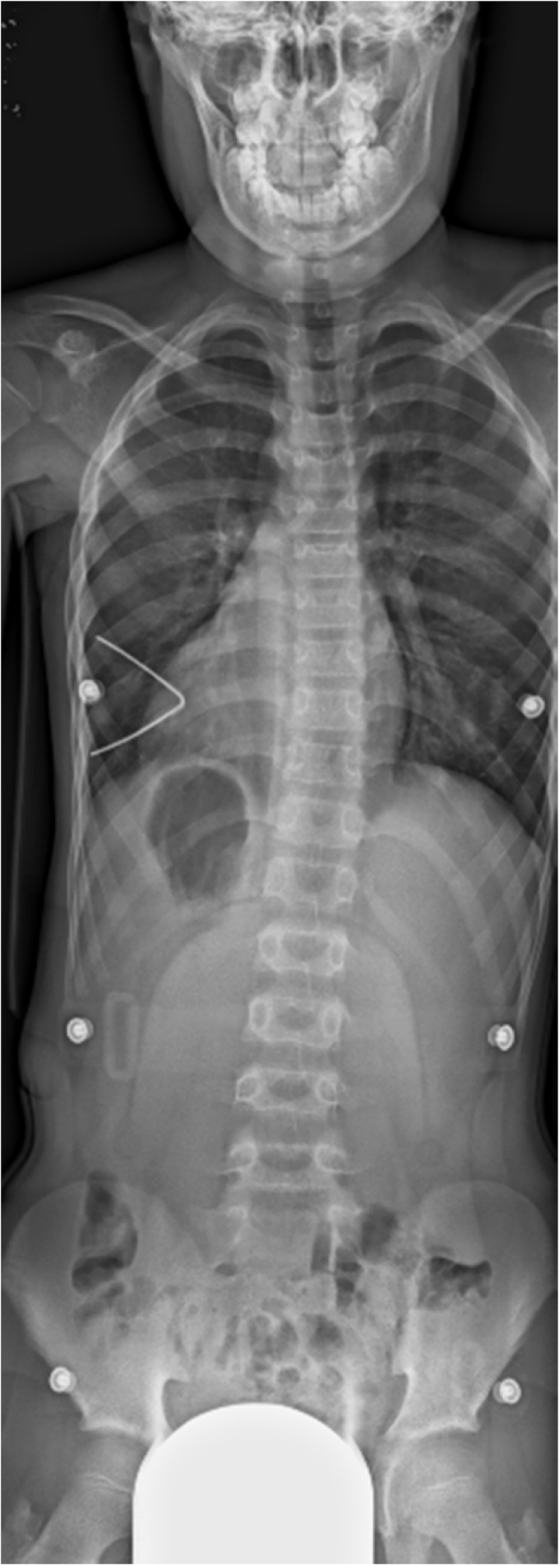
Radiograph wearing underarm brace. At treatment commencement, in spite of slight overcorrection, sufficient scoliotic curve correction by the brace was verified

**Fig. 4:**
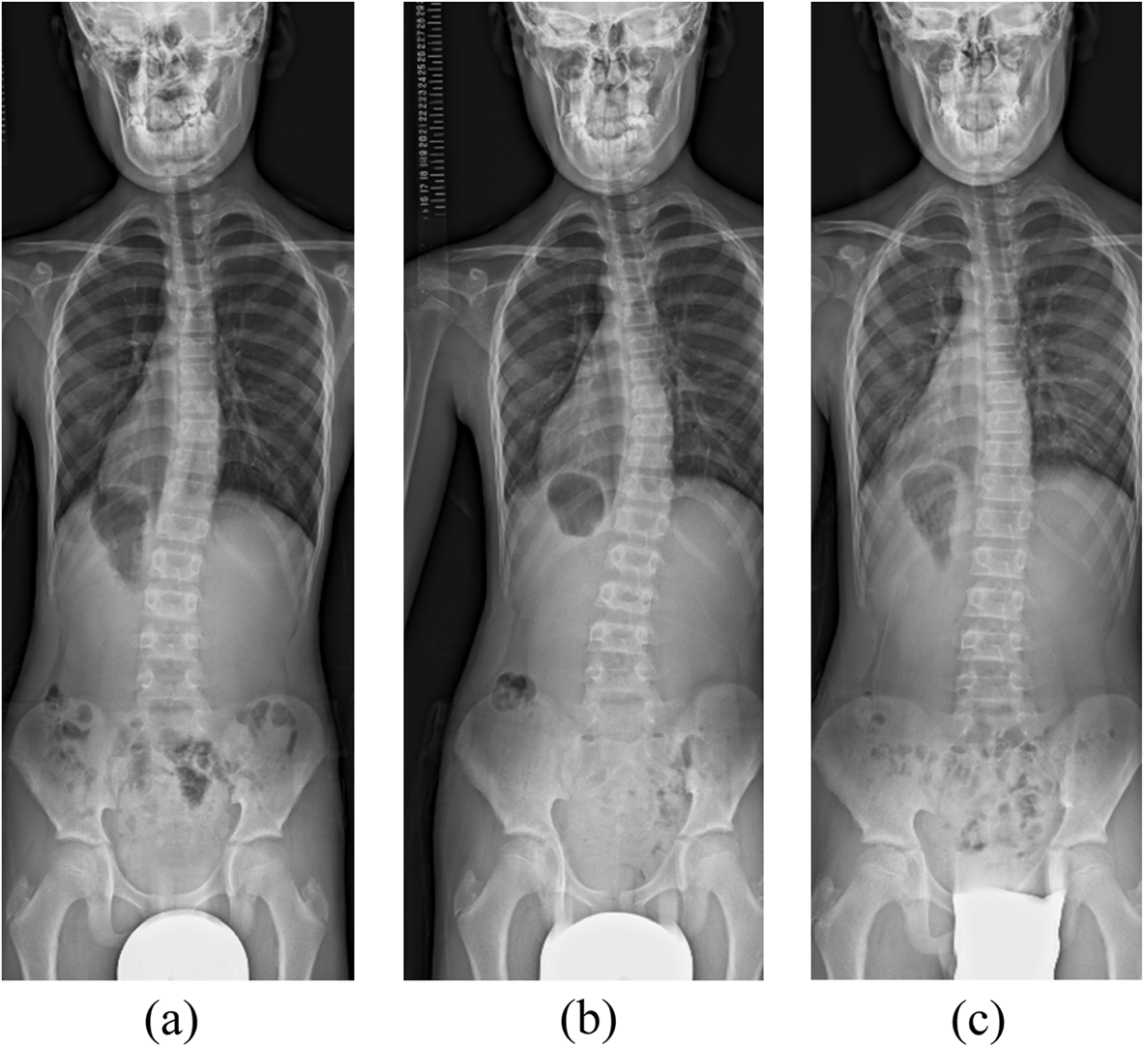
Radiographs during brace treatment. **a** At 3 months brace treatment, the scoliotic curve had improved to 16 degrees (T5-L2), although the curve had reversed into a right convex curve. **b** Scoliotic angle had increased to 21 degrees (T5-L2) at 7 months of brace treatment. **c** After another adjustment of the brace, his right convex scoliotic angle improved to 13 degrees (T4-L2) at 15 months of treatment

A 4-year-old boy was diagnosed as having Goldenhar syndrome and referred to our hospital for scoliosis treatment. His scoliotic angle was 5 degrees (T10-L3) with Risser grade 0 (Fig. 1a). His spinal curve deteriorated gradually, and left convex scoliotic angle was increased at 26 degrees (T3-L2) with Risser grade 0 and angle of trunk rotation (ATR) of 5 degrees at 11 years of age to necessitate the prescription of an underarm brace (Fig. 1b). Whole-spine MRI revealed no abnormalities, such as Chiari malformations, syringomyelia, spinal cord cavities, spinal cord tumors, dural ectasia, and low-level conus medullaris (Fig. 2). At treatment commencement, in spite of slight overcorrection, the brace was confirmed to sufficiently correct the scoliotic curve (Fig. 3). Although he was instructed to wear the brace for at least 16 h daily, the reported mean wearing time was approximately 10 h. At 3 months of treatment, his scoliotic curve was improved at 16 degrees (T5-L2) with Risser grade 0. However, the deformity had reversed into a right convex curve (Fig. 4a). In spite of wearing the brace more loosely, his right convex scoliotic angle had increased to 21 degrees (T5-L2) with Risser grade 0 and ATR of 3 degrees at 7 months of treatment (Fig. 4b). After another adjustment of the brace, his right convex scoliotic angle improved to 13 degrees (T4-L2) with Risser grade 0 and ATR of 2 degrees at 15 months of treatment (Fig. 4c).

## Discussion and conclusions

Goldenhar syndrome is a rare condition that is often accompanied with progressive scoliosis [4–6]. In a report of 35 patients with Goldenhar syndrome, 60% had vertebral anomalies, most of which exhibiting a congenital vertebral abnormality such as hemivertebra [7]. Tsirikos et al. reviewed 668 cases of congenital spinal deformity and identified a 2% prevalence of hemifacial microsomia [8]. Of these patients, 71.5% also exhibited congenital thoracic scoliosis [8]. McKay et al. described scoliosis, spina bifida, vertebral fusion, hemivertebra, and rib anomalies as the major vertebral anomalies in Goldenhar syndrome [9]. Spinal surgery at an early stage is frequently necessary for patients with congenital spinal deformities to balance spine growth and prevent progressive deformity according to the indications for congenital scoliosis surgery described by Winter and McMaster et al. [10–13]. In the present case of Goldenhar syndrome, the scoliotic deformity was progressive but Cobb angle was not severe. Furthermore, the deformity was not congenital and no hemivertebra or vertebral abnormalities were detected. Thus, brace treatment was prescribed to prevent the advancement of scoliosis.

The efficacy of brace treatment for progressive scoliosis has been described [14–16]. On the other hand, few reports have documented overcorrection using this method; Hohman et al. reported one case of adolescent idiopathic scoliosis with overcorrection due to brace treatment as a very rare phenomenon [17]. In the present patient, the scoliotic curve could be corrected sufficiently by the brace, but the curve reversed at 3 months of treatment. Furthermore, the reversed curve progressed despite loosening the brace. Although this appears to be a very rare outcome, clinicians may opt to periodically check curve correction despite the risk of increased radiation exposure. Alternatively, halting brace treatment at 3 months of treatment is possible. However, since curve progression after brace weaning has also been reported [18], such a decision may be difficult. As this is a very rare case, continued observation is necessary due to the short follow-up period and patient’s skeletal immaturity.

One limitation of this report was that we were unable to determine whether the patient’s scoliosis was constructive. However, since ATR measurements indicated rotation of the spine, it was likely that the deformity could not be explained solely by postural effects. The scoliosis characteristic of Goldenhar syndrome is congenital and often associated with abnormalities of the maxillofacial skeleton, ribs, and vertebrae [7–9]. As the present case displayed relatively mild scoliosis without the above anomalies, it could be considered to resemble a simple case of juvenile type idiopathic scoliosis. Thus, the curve inversion in this patient may be a common idiopathic scoliosis problem as well.

## Data Availability

The datasets used and/or analysed during the current study are available from the corresponding author on reasonable request.
